# Mortality Outcomes in Dermatology: An Exploration of Core Outcome Sets and Cochrane Skin Systematic Reviews

**DOI:** 10.2196/34140

**Published:** 2022-02-01

**Authors:** Torunn E Sivesind, Mindy D Szeto, Shahzeb Hassan, Peter Tugwell, Robert P Dellavalle

**Affiliations:** 1 Department of Dermatology University of Colorado Anschutz Medical Campus Aurora, CO United States; 2 Feinberg School of Medicine Northwestern University Chicago, IL United States; 3 Department of Medicine School of Epidemiology and Public Health University of Ottawa Ottawa, ON Canada

**Keywords:** mortality, death, systematic reviews, outcomes, dermatology, Cochrane

Cochrane has been a trusted proponent of evidence-based medicine for over 20 years. Its dermatology-specific editorial team (Cochrane Skin Review Group) is the pre-eminent source of systematic reviews in dermatology [1]. Explicit standardized Cochrane review methods can minimize bias and maximize the reliability of reported outcomes, establishing benchmarks for decision-making. Mortality is one outcome where pronounced heterogeneity in reporting may affect its utility in clinical research. We therefore explored mortality outcome expression and execution in the Cochrane Skin portfolio and concurrently analyzed mortality in core outcome sets (outcomes that, at a minimum, should be measured in clinical trials) by searching dermatology studies registered in the COMET (Core Outcome Measures in Effectiveness Trials) database [2]. COMET contains text from core outcome sets publications, from which we extracted core outcomes and classified these according to the taxonomy developed by Dodd et al [3] for validated standardized annotation.

All Cochrane Skin Group reviews as of March 2021 were included and exported from the Cochrane Database of Systematic Reviews [1], allowing descriptive analysis and characterization of mortality reporting by category of mortality terminology (all-cause, cause-specific, infant/maternal, survival). All COMET database core outcome sets classified in the published “skin” research category as of August 23, 2021, were reviewed for reporting of mortality outcomes and categorized according to the mortality terminology previously described. Core outcomes specified in terms of “death” were included in the all-cause mortality category.

Of the 113 Cochrane Skin dermatology reviews, 13 reported mortalities as an outcome measure: 10 all-cause, 2 cause-specific, 5 survival, and 1 infant/maternal (Table 1).

Four reviews (4/13) reported more than one mortality outcome. More than one-third of the total reviews (5/13) were melanoma-related. Reviews of other dermatologic conditions reporting mortality included cutaneous squamous cell carcinoma (cSCC), nonmelanoma skin cancer, pemphigus vulgaris, pemphigus foliaceus, bullous pemphigoid, toxic epidermal necrolysis (TEN), necrotizing fasciitis, drug-induced skin rash, and topical steroids used during pregnancy. The time frame of mortality outcome reporting ranged widely, from 10 days to 10 years, but generally correlated appropriately with the condition (eg, 30 days for TEN capturing acute onset and progression vs 10-year survival for melanoma).

COMET database searches revealed 13 core outcome set studies of 13 skin conditions (Table 2); only 2 (15%) included mortality as a core outcome (survival for head and neck lymphatic malformations, death from cSCC).

**Table 1:** Mortality reporting in Cochrane Skin Systematic Reviews as of March 2021.

Condition	Cochrane Systematic Review title	Authors	Year	DOI	PMID	Type of mortality reported	Time frame of mortality reporting
Toxic epidermal necrolysis	Interventions for Toxic Epidermal Necrolysis	Majumdar S, Mockenhaupt M, Roujeau J, Townshend A	2002	10.1002/14651858.CD001435	12519556	All-cause mortality	30-day follow-up time
Melanoma	Statins and Fibrates for Preventing Melanoma	Dellavalle RP, Drake A, Graber M, Heilig LF, Hester EJ, Johnson KR, McNealy K, Schilling L	2005	10.1002/14651858.CD003697.pub2	16235336	Disease-specific	≥7 years post-RCT^a^
Nonmelanoma skin cancers	Interventions for Preventing Nonmelanoma Skin Cancers in High-Risk Groups	Bath-Hextall F, Leonardi-Bee J, Somchand N, Webster A, Delitt J, Perkins W	2007	10.1002/14651858.CD005414.pub2	17943854	All-cause mortality	End of trial follow-up (1 year to 5 years for included RCTs)
Pemphigus vulgaris and pemphigus foliaceus	Interventions for Pemphigus Vulgaris and Pemphigus Foliaceus	Martin LK, Agero AL, Werth V, Villanueva E, Segall J, Murrell DF	2009	10.1002/14651858.CD006263.pub2	19160272	All-cause mortality	Variable, deaths only reported from 1 RCT over 4 weeks
Melanoma	Surgical Excision Margins for Primary Cutaneous Melanoma	Sladden MJ, Balch C, Barzilai DA, Berg D, Freiman A, Handiside T, Hollis S, Lens MB, Thompson JF	2009	10.1002/14651858.CD004835.pub2	19821334	All-cause mortality, survival, recurrence-free survival	5- and 10-year survival
Bullous pemphigoid	Interventions for Bullous Pemphigoid	Kirtschig G, Middleton P, Bennett C, Murrell DF, Wojnarowska F, Khumalo NP	2010	10.1002/14651858.CD002292.pub3	20927731	All-cause mortality	51 days (1 RCT), 10 days (1 RCT), 6 months and 3 years (1 RCT)
Cutaneous squamous cell carcinoma	Interventions for Nonmetastatic Squamous Cell Carcinoma of the Skin	Lansbury L, Leonardi-Bee J, Perkins W, Goodacre T, Tweed JA, Bath-Hextall FJ	2010	10.1002/14651858.CD007869.pub2	20393962	All-cause mortality	2 years
Melanoma	Interferon Alpha for the Adjuvant Treatment of Cutaneous Melanoma	Mocellin S, Lens MB, Pasquali S, Pilati P, Chiarion Sileni V	2013	10.1002/14651858.CD008955.pub2	23775773	Death, disease-free survival, overall survival	5 years
Melanoma	Sentinel Lymph Node Biopsy Followed by Lymph Node Dissection for Localized Primary Cutaneous Melanoma	Kyrgidis A, Tzellos T, Mocellin S, Apalla Z, Lallas A, Pilati P, Stratigos A	2015	10.1002/14651858.CD010307.pub2	25978975	All-cause mortality, disease-specific, disease-free survival	10 years
Pregnancy	Safety of Topical Corticosteroids in Pregnancy	Chi CC, Wang SH, Wojnarowska F, Kirtschig G, Davies E, Bennett C	2015	10.1002/14651858.CD007346.pub3	26497573	Fetal death	Not specified, variable
Necrotizing soft tissue infections	Interventions for Necrotizing Soft Tissue Infections in Adults	Hua C, Bosc R, Sbidian E, De Prost N, Hughes C, Jabre P, Chosidow O, Le Cleach L	2018	10.1002/14651858.CD011680.pub2	29851032	Mortality, survival	30-day mortality, 28-day and 30-day study periods for survival
Melanoma	Systemic Treatments for Metastatic Cutaneous Melanoma	Pasquali S, Hadjinicolaou AV, Chiarion Sileni V, Rossi CR, Mocellin S	2018	10.1002/14651858.CD011123.pub2	29405038	Overall survival, progression-free survival	1 year
Severe drug‐induced skin rash	Genetic Testing for Prevention of Severe Drug‐Induced Skin Rash	Alfirevic A, Pirmohamed M, Marinovic B, Harcourt-Smith L, Jorgensen AL, Cooper TE	2019	10.1002/14651858.CD010891.pub2	31314143	All-cause mortality	12-month follow-up post rash

^a^RCT: randomized controlled trial.

**Table 2:** Mortality as an Outcome in COMET (Core Outcome Measures in Effectiveness Trials).

Condition	Study title	Authors	Year	URL	DOI	Mortality as an outcome (yes/no)	Type of mortality reported
Acne	Identifying What to Measure in Acne Clinical Trials: First Steps Towards Development of a Core Outcome Set	Layton AM, et al	2017	http://www.comet-initiative.org/Studies/Details/1221	http://dx.doi.org/10.1016/j.jid.2017.04.017	No	N/A^a^
Actinic keratosis	Core Outcome Set for Actinic Keratosis Clinical Trials	Reynolds KA, et al	2019	http://www.comet-initiative.org/Studies/Details/756	http://dx.doi.org/10.1001/jamadermatol.2019.4212	No	N/A
Cutaneous leishmaniasis	Harmonized Clinical Trial Methodologies for Localized Cutaneous Leishmaniasis and Potential for Extensive Network With Capacities for Clinical Evaluation	Olliaro P, et al	2018	http://www.comet-initiative.org/Studies/Details/1455	https://doi.org/10.1371/journal.pntd.0006141	No	N/A
Eczema	Core Outcome Domains for Controlled Trials and Clinical Recordkeeping in Eczema: International Multiperspective Delphi Consensus Process	Schmitt J, et al	2011	https://www.comet-initiative.org/Studies/Details/90	http://dx.doi.org/doi:10.1038/jid.2010.303	No	N/A
Head and neck lymphatic malformation	Standardized Outcome and Reporting Measures in Pediatric Head and Neck Lymphatic Malformations	Balakrishnan K, et al	2015	http://www.comet-initiative.org/Studies/Details/894	https://doi.org/10.1177/0194599815577602	Yes	Death
Hidradenitis suppurativa	A Core Domain Set for Hidradenitis Suppurativa Trial Outcomes: An International Delphi Process	Thorlacius L, et al	2018	http://www.comet-initiative.org/Studies/Details/934	http://dx.doi.org/10.1111/bjd.16672	No	N/A
Incontinence-associated dermatitis	Core Outcome Domains in Incontinence-Associated Dermatitis Research	Van den Bussche K, et al	2018	http://www.comet-initiative.org/Studies/Details/383	http://dx.doi.org/10.1111/jan.13562	No	N/A
Psoriasis	Identifying a Core Domain Set to Assess Psoriasis in Clinical Trials	Callis Duffin K, et al	2018	http://www.comet-initiative.org/Studies/Details/1464	Not available	No	N/A
Skin cancer	Development of a Core Outcome Set for Cutaneous Squamous Cell Carcinoma Trials: Identification of Core Domains and Outcomes	Reynolds KA, et al	2020	http://www.comet-initiative.org/Studies/Details/864	http://dx.doi.org/10.1111/bjd.19693	Yes	Progression-free survival, recurrence-free survival, disease-specific survival
Vascular malformations	Development of an International Core Outcome Set for Peripheral Vascular Malformations (OVAMA Project)	Horbach SER, et al	2018	http://www.comet-initiative.org/Studies/Details/767	http://dx.doi.org/10.1111/bjd.16029	No	N/A
Vasculitis (small-vessel/ ANCA^b^-associated)	Clinicians’ Perspective on Key Domains in ANCA-Associated Vasculitis: a Delphi Exercise	Milman N, et al	2017	http://www.comet-initiative.org/Studies/Details/1041	http://dx.doi.org/10.1080/03009742.2016.1188980	No	Death discussed (from OMERACT^c^, to which this study adds—but was not directly included in this study)
Vitiligo	Developing Core Outcome Set for Vitiligo Clinical Trials: International e-Delphi Consensus	Eleftheriadou V, et al	2015	http://www.comet-initiative.org/Studies/Details/357	http://dx.doi.org/10.1111/pcmr.12354	No	N/A
Vulval skin disorders	Outcome Measures for Vulval Skin Conditions: a Systematic Review of Randomised Controlled Trials	Simpson R, et al	2013	https://www.comet-initiative.org/Studies/Details/271	http://dx.doi.org/DOI:%2010.1111/bjd.12391	No	N/A

^a^N/A: not applicable.

^b^ANCA: antineutrophil cytoplasmic autoantibody.

^c^OMERACT: Outcome Measures in Rheumatoid Arthritis Clinical Trials.

Although limited in the number of studies appraised, our results illustrate substantial variability in the reporting and timing of mortality outcomes in Cochrane Skin reviews and COMET dermatology-related core outcome sets. Allowance of potentially unclear metrics (eg, “death”) and fluctuations in the time frame considered (especially within studies of a particular disease) may be detrimental to the downstream harmonization and generalizability of research findings. Guidelines to assist researchers during trial design and registration would encourage the selection of clear metrics and facilitate consistent outcome reporting at the later stages. Increased guidance and communication among stakeholders in this area, including further refinement of reporting guideline statements such as CONSORT (Consolidated Standards of Reporting Trials) [4] and PRISMA (Preferred Reporting Items for Systematic Reviews and Meta-Analyses) [5], could promote much-needed standardization in mortality reporting, facilitating comparison across studies and helping decision makers effectively use dermatology research.

## Abbreviations

COMET: Core Outcome Measures in Effectiveness Trials
CONSORT: Consolidated Standards of Reporting Trials
cSCC: cutaneous squamous cell carcinoma
JAAD: Journal of the American Academy of Dermatology
JID: Journal of Investigative Dermatology
PRISMA: Preferred Reporting Items for Systematic Reviews and Meta-Analyses
TEN: toxic epidermal necrolysis
